# In *Silico* and Biochemical Analysis of *Physcomitrella patens* Photosynthetic Antenna: Identification of Subunits which Evolved upon Land Adaptation

**DOI:** 10.1371/journal.pone.0002033

**Published:** 2008-04-30

**Authors:** Alessandro Alboresi, Stefano Caffarri, Fabien Nogue, Roberto Bassi, Tomas Morosinotto

**Affiliations:** 1 Laboratoire de Génétique et Biophysique des Plantes-UMR 6191 CEA-CNRS-Université de la Méditerranée, Marseille, France; 2 Station de Génétique et Amélioration des Plantes, INRA, IJPB, Versailles, France; 3 Dipartimento Scientifico e Tecnologico, Università di Verona, Verona, Italy; 4 Dipartimento di Biologia, Università di Padova, Padova, Italy; National Institute on Aging, United States of America

## Abstract

**Background:**

In eukaryotes the photosynthetic antenna system is composed of subunits encoded by the light harvesting complex (Lhc) multigene family. These proteins play a key role in photosynthesis and are involved in both light harvesting and photoprotection. The moss *Physcomitrella patens* is a member of a lineage that diverged from seed plants early after land colonization and therefore by studying this organism, we may gain insight into adaptations to the aerial environment.

**Principal Findings:**

In this study, we characterized the antenna protein multigene family in *Physcomitrella patens*, by sequence analysis as well as biochemical and functional investigations. Sequence identification and analysis showed that some antenna polypeptides, such as Lhcb3 and Lhcb6, are present only in land organisms, suggesting they play a role in adaptation to the sub-aerial environment. Our functional analysis which showed that photo-protective mechanisms in *Physcomitrella patens* are very similar to those in seed plants fits with this hypothesis. In particular, *Physcomitrella patens* also activates Non Photochemical Quenching upon illumination, consistent with the detection of an ortholog of the PsbS protein. As a further adaptation to terrestrial conditions, the content of Photosystem I low energy absorbing chlorophylls also increased, as demonstrated by differences in Lhca3 and Lhca4 polypeptide sequences, *in vitro* reconstitution experiments and low temperature fluorescence spectra.

**Conclusions:**

This study highlights the role of Lhc family members in environmental adaptation and allowed proteins associated with mechanisms of stress resistance to be identified within this large family.

## Introduction

Photosynthesis is powered by light absorbed by chlorophyll (Chl^1^) and carotenoid molecules bound to thylakoid membrane proteins. Pigment-binding proteins are organized into two supramolecular complexes: Photosystem (PS) I and II. Each Photosystem is composed of two different moieties: (i) the core complex, responsible for charge separation and the first steps of electron transport, and (ii) the peripheral antenna system, which plays a role in light harvesting and the transfer of excitation energy to the reaction center. Reaction centers are widely conserved among organisms and only small differences are found between seed plants and cyanobacteria whereas antenna systems, in contrast, are more variable [1], [2]. In most prokaryotes the antenna system is based on phycobilisomes while in eukaryotes it is composed of members of a multigene family which in broad terms is represented by Chl a/b-xanthophyll binding proteins in seed plants and green algae, Carotenoid-Chl a/c proteins in chromophytes and the intrinsic peridinin-Chl a/c proteins (Lhcd) present in many dinoflagellates [1], [3], [4]. Despite diverse pigment content, primary sequence similarities indicate that all these antenna proteins originated from a common ancestral gene with one transmembrane helix [1]. In green algae and land plants, which are the focus of this work, the antenna system is composed of Light Harvesting Complex (Lhc) proteins which have three transmembrane helices and bind Chl a, b and xanthophylls [1]. Two sub-classes of Lhc proteins are preferentially associated with PSI or PSII and are defined as Lhca and Lhcb, respectively [5]. In *Arabidopsis thaliana*, six different polypeptides were identified for PSI (Lhca1-6) and eight for PSII (Lhcb1-8), including the recently proposed additions, Lhcb7 and Lhcb8 [5], [6].

As mentioned above, antenna proteins play a role in increasing light harvesting and energy transfer to reaction centers. However, light is not always a limiting factor and photosynthetic organisms are often exposed to conditions where absorbed energy exceeds their capacity for electron transport [7]. The presence of excess energy in the form of ^1^Chl* excited states is dangerous for the cell, since ^3^Chl*, produced by intersystem crossing, can easily react with molecular oxygen and produce reactive species, especially singlet oxygen [7], [8]. Several photoprotection mechanisms have evolved to prevent/reduce the damage caused by excess energy [8], [9]. Although these mechanisms appear to be interdependent and overlapping they lead to photoprotection on different time scales. The fastest photoprotection mechanism is called Non Photochemical Quenching (NPQ) which consists of thermal dissipation of the excess ^1^Chl* states. NPQ is activated within seconds of a change in light intensity and its action was proven to be of particular importance under rapidly changing light conditions [10]. The fastest NPQ component is dependent on lumen acidification and the presence of a Lhc-like protein, PsbS [11]. On a minutes time scale after the onset of a light stress, zeaxanthin is synthesized from violaxanthin, in a process known as the xanthophyll cycle [9]. Zeaxanthin is a carotenoid with multiple roles in photoprotection: it enhances NPQ itself [12], it establishes a long term quenching effect by binding to Lhc proteins [13] and it contributes to ROS scavenging [14]. On a longer time scale, photosynthetic organisms acclimate to light regimes by activating several responses such as antenna size regulation and modulation of metabolic sinks [15]. Members of the Lhc multigene family are involved in all these photoprotection mechanisms, regardless of their activation time scale. All proteins do not have equivalent functions, however, and play prominent roles either in light harvesting or photoprotection [16].

In organisms belonging to the Streptophyta, other polypeptides show significant homology to Lhc proteins. These have a different structural organization to Lhc, with a variable number of transmembrane helices but share an evolutionary origin from a single helix ancestor [1]. These gene products were proposed to be specialized in photoprotection and adaptation to different light environments [1], a hypothesis well supported by experimental data in the case of PsbS [11] but still under debate for ELIPs [17]–[19].

Research on the Lhc multigene family has focused mainly on seed plants and, secondarily, on algae (green, brown, red algae, diatoms). Little information is available on bryophytes (mosses, liverworts, hornworts), which diverged from the ancestor of seed plants more than 400 million years ago, during the first phase of colonization of the aerial environment [20]. As a lineage, mosses are a crucial group for understanding the transition to life on land: most moss vegetative tissues are haploid like their green algal ancestors, but the needle-like shoots that produce spores (sporophytes) display key innovations for life outside water, such as stomata and simple strands of conductive cells [21], [22]. The analysis of bryophytes, thus, could help identify genes which evolved early after the transition from aquatic to terrestrial life. This is of particular interest for photosynthetic organisms because new challenges were associated with sub-aerial life such as direct illumination and fast O_2_ diffusion, all factors causing enhanced photo-oxidative stress, a major limitation for plant growth.

In this study, we characterized the antenna proteins in the moss *Physcomitrella patens*, by analyzing their sequence, their biochemical properties and function. We first identified the Lhc and Lhc-like polypeptides of this organism by exploiting EST databases and the recently completed genome sequence [22]–[25]. By comparison with antenna proteins from other green lineage organisms, we showed that some Lhc polypeptides, notably Lhcb3 and Lhcb6, are detectable only in land plants, suggesting they may play a role in adaptation to aerial environments. This hypothesis is consistent with other physiological adaptations by *Physcomitrella patens*: this moss showed a capacity for activating Lhc-dependent mechanisms in response to environmental conditions in a similar fashion to seed plants. One major difference with seed plants was that an Lhca4 ortholog was absent in *P. patens*. By analyzing low temperature fluorescence spectra, we showed that the lack of Lhca4 is consistent with the content of red-shifted spectral forms observed in moss Photosystem I.

## Results

### Identification and classification of the Lhc sequence family in Physcomitrella patens

Light harvesting proteins (Lhc) are members of a large multigene family and play an important role in the light phase of photosynthesis and response to environmental conditions. With the aim of characterizing this family in *Physcomitrella patens*, we first exploited the available sequence databases to identify moss Lhc family members in the genome.

We thus searched for contig sequences with significant homology to *Arabidopsis thaliana* and *Chlamydomonas reinhardtii* Lhc polypeptides in the *P. patens* EST database (Physcobase, http://moss.nibb.ac.jp/). In cases where a positive result was obtained, we carried out further searches to identify all the EST clones corresponding to the contig, assuming that the number of clones gives an idea of the level of gene expression. In a few cases, clones without a corresponding contig were identified and assembled from individual sequences. Sequencing of the entire *Physcomitrella* genome was completed very recently and it was used to confirm the EST sequences [22], [25]. In most cases, the sequences retrieved from the two databases were identical; however, by using this additional information we were also able to identify a few new genes, as reported below. The first result obtained from is that the Lhc multigene family contains more members in *Physcomitrella* than *Arabidopsis*. The presence of a large number of Lhc polypeptides in *Physcomitrella* was suggested by a previous study [26] and is likely due to recent duplication of part of the *Physcomitrella* genome [27].

In order to classify the *P. patens* Lhc proteins, sequences retrieved were compared to sequences from other organisms belonging to the green lineage. In this analysis we included some fully sequenced organisms which diverged at different times during evolution: i) *Ostreococcus tauri*, which is the smallest free-living eukaryote identified to date and belongs to the Prasinophyceae class, one of the most ancient clade among Chlorophyta [28]. ii) *C. reinhardtii*, a model organism for photosynthetic unicellular eukaryotes, also belonging to Chlorophyta [29]; iii-iv) *A. thaliana* and *Populus spp.*, as model organisms for dicotyledons, and v) *Oryza sativa* for monocots. Lhc protein sequences from these organisms were obtained from previous studies [5], [6], [30]–[32]. We also included sequences retrieved from EST databases representing several different algae species: among these *Mesostigma viride* is worth mentioning because it is an alga belonging to Streptophyta [33]–[35], and thus diverged from land plants ancestor later than Chlorophyta.

As expected, the sequence alignment showed that Lhc proteins from *Physcomitrella* are more similar to plant genes than to algal counterparts. Therefore, when possible, the *P. patens* nomenclature followed that of the nearest *Arabidopsis* gene, as reported in supplementary figure S1. When multiple isoforms of the same gene were present, they were numbered starting from the isoform showing the highest number of EST clones in the database, assuming their number is proportional to gene expression level.

It was not always clearly straightforward to assign a direct ortholog relationship between *P. patens* and *Arabidopsis* genes. For the major antenna complex of Photosystem II (LHCII), several proteins showed high similarity to *Arabidopsis* Lhcb1 and Lhcb2 but it was not possible to find a direct correspondence to a specific *Arabidopsis* isoform. To avoid any confusion with the cases where a direct ortholog was found, constituents of the LHCII major antenna complex were named Lhcbm, according to [32], [36].

No *Physcomitrella* orthologs of AtLhca4 and AtLhca6 (At, *A. thaliana*) were found. In the case of AtLhca5, an ortholog was found in the genome database but not among EST sequences, suggesting that, in *Physcomitrella,* Lhca5 is not expressed or only in special growth conditions different from those used for EST library construction. This is a peculiarity of this subunit, since all other *Lhc* sequences present in the genome database were highly represented in expressed sequence libraries.

Interestingly, we also identified a new Lhc protein specific to *Physcomitrella*. Since this sequence shows significant similarity to Lhcb polypeptides, it was tentatively attributed to the PSII antenna system and named Lhcb9.

### Phylogenetic tree of Photosystem I antenna proteins

**Figure 1:**
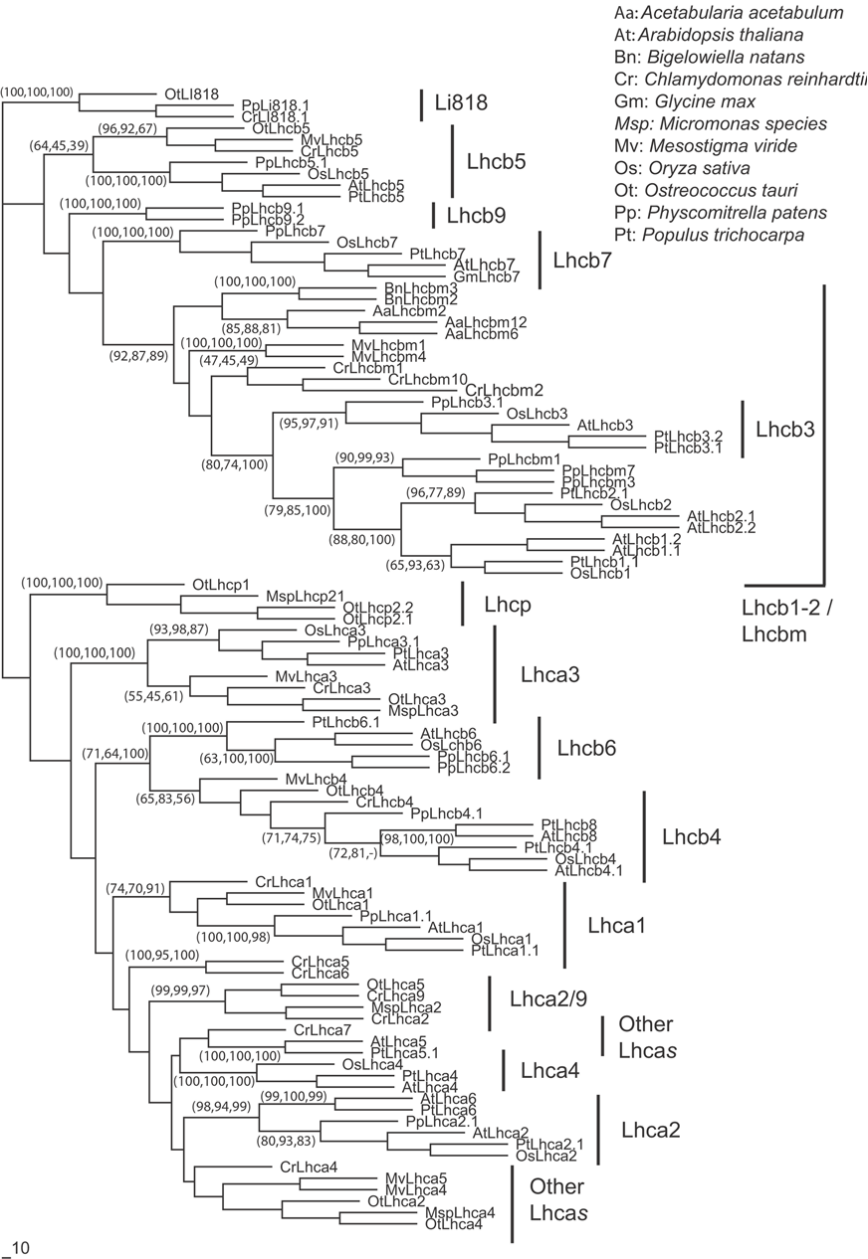
Phylogenetic tree of Lhc. Phylogenetic tree of all Lhc proteins identified in *A. thaliana* (At), *Populus trichocarpa* (Pt), *O. sativa* (Os), *P. patens* (Pp), *C. reinhardtii* (Cr) and *O. tauri* (Ot). Sequences obtained from several algae species EST databases were also included (*Acetabularia acetabulum,* Aa, *Mesostigma viride,* Mv, *Micromonas sp.,* Msp, *Bigelowiella natans*, Bn). A total of 100 sequences was analyzed. Four Li818 sequences from *P. patens* (Pp), *C. reinhardtii* (Cr) and *O. tauri* (Ot) were also included as an external out-group. The tree was built using three different approaches: here we show the results from the maximum likelihood approach. The bootstrap values obtained from maximum likelihood, NJD and maximum parsimony approaches respectively are shown in the tree nodes. For clarity, bootstrap values are not shown when consistency was poor and when nodes are not useful to discriminate isoforms.

Since all Lhc proteins share a certain degree of similarity, in order to accurately assign *Physcomitrella* polypeptides to groups of corresponding isoforms from other plants, we generated phylogenetic trees (for an example of sequence alignments and the list of sequences included see supplemental figure S2 and S3). We employed different tree calculation algorithms and the tree topology was dependent on the algorithm employed; nevertheless, some features were consistently present in different analyses. In particular, we always found specific clusters of different Lhc isoforms, as shown in figure 1.

Within the PSI antenna system, proteins are generally more difficult to classify into different isoform groups since they appear to diverge more than Lhcb polypeptides, as previously observed in the case of *Chlamydomonas* [31]. For this reason, we confirmed the analysis in figure 1 by producing another set of trees with an increased number of Lhca sequences, which is reported in additional materials (Fig. S4). In both cases we clearly found that at least one Lhca1 and Lhca3 isoform is conserved in all the fully sequenced species included in the study: this conservation was especially remarkable for Lhca3 with small sequence differences observed between *A. thaliana, P. patens, C. reinhardtii* and *O. tauri*. Four different AtLhca2 orthologs were found in *Physcomitrella* and these clustered with other plant Lhca2 isoforms. In the case of algae, we detected several sequences with significant similarity to Lhca2 [31]. Although the degree of similarity was clearly lower with respect to Lhca1 and Lhca3, at least one Lhca2-like polypeptide was present in all six fully sequenced organisms. We could not find obvious AtLhca4 and AtLhca6 orthologs in *P. patens* in either the EST or genomic databases. From the tree in figure 1, AtLhca6 can be classified as a Lhca2-like protein, as previously suggested [5]. AtLhca4 also shares some similarity with Lhca2 but orthologs of this polypeptide are found only in other seed plants (both monocots and dicots) and not in the other species analyzed here, including in *P. patens*. When we searched other non-seed plant EST databases, however, we found sequences identifiable as Lhca4 even if only in *Selaginella moellendorffii* and *Marchantia polymorpha.*

With regards to the remaining PSI antenna polypeptide, Lhca5, it was identified in *Arabidopsis* and other mono- and dicot seed plants [37] and a gene encoding a homologous polypeptide was also found in *Physcomitrella*. Sequences annotated as Lhca5 are also found in *Chlamydomonas* and *O. tauri* but their conservation is too low to support the hypothesis of a conserved Lhc isoform.

Interestingly, we also identified a Lhca sub-group containing Lhca2/9 genes specifically from algae, as recently suggested [32]. These polypeptides, however, were not conserved during evolution of land plants or evolved in green algae after their separation from land plants and, despite their name, have no particular similarity with plant Lhca2.

### Phylogenetic tree of Photosystem II antenna proteins

Among the sequences analyzed, several polypeptides were identified as LHCII components and named Lhcb1-2-3 or Lhcbm, depending on the species. These isoforms, however, were not found in *O. tauri*, where the major antenna complex is composed of Lhcp polypeptides, which were also identified in other prasinophytes such as *Mantoniella squamata* [38] and in *Mesostigma viride* [32].

**Figure 2:**
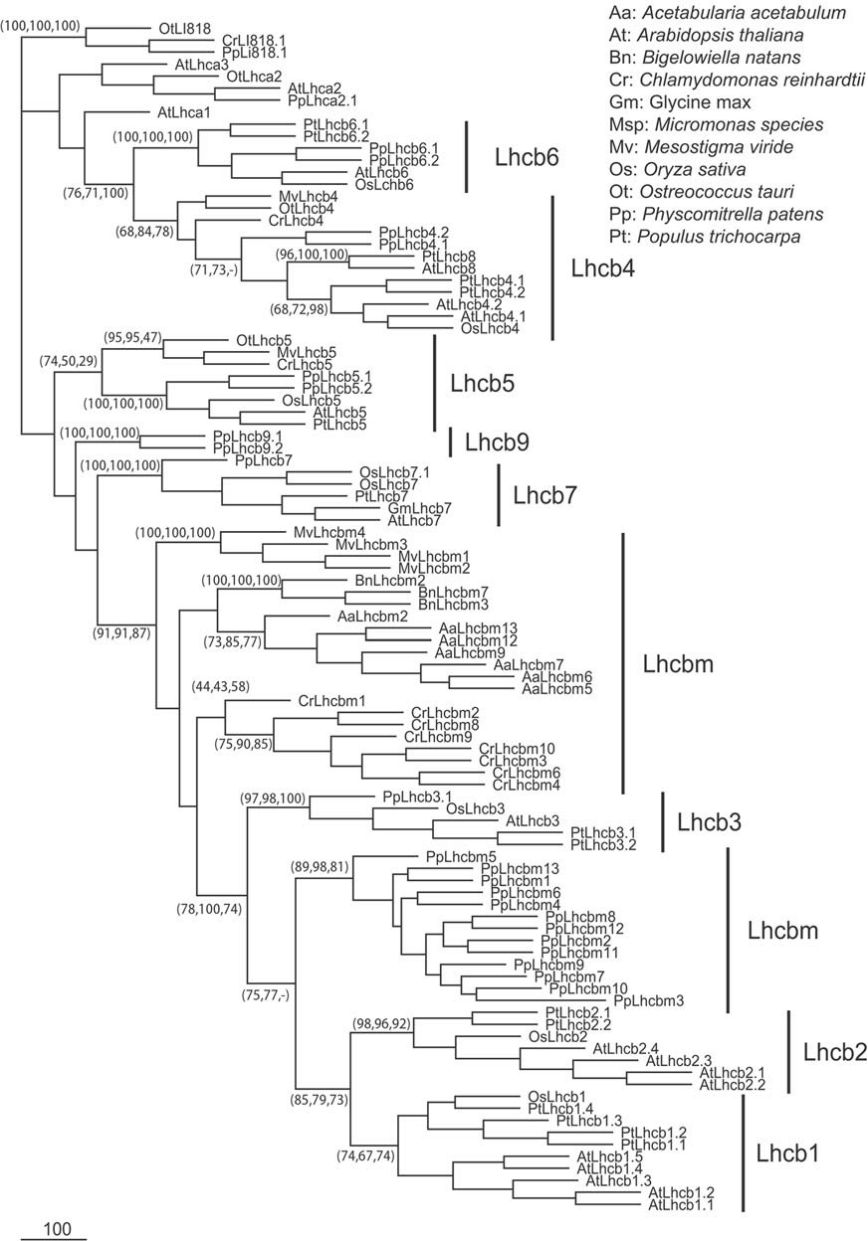
Phylogenetic tree of proteins composing the major PSII antenna complex. A Phylogenetic tree was generated as described for figure 1. In this case, more PSII and less PSI sequences were included but a total of 100 sequences were analyzed. The four Li818 sequences from *P. patens* (Pp), *C. reinhardtii* (Cr) and *O. tauri* (Ot) were included once again as an external out-group. The tree shown was constructed using a maximum likelihood approach, but the bootstrap values shown in tree nodes were obtained from maximum likelihood, NJD and maximum parsimony approaches respectively. For clarity, bootstrap values are not shown when consistency was poor and when nodes are not useful to discriminate isoforms.

For the LHCII components, several gene copies encoding highly similar polypeptides were found for each species. A phylogenetic tree where all these multiple copies were included is shown in figure 2: this tree clearly shows that these sequences diverged after the separation of each species. The distinction of Lhcb1 and Lhcb2 isoforms is found only in monocot and dicot seed plants [5], [39], but is not conserved in *Physcomitrella* or in any algae species analyzed. This was confirmed by detailed analysis of sequence alignments: in highly conserved regions (e.g. helix B shown in Fig. S6), the small differences identified are consistently maintained among proteins of the same species, suggesting that all copies evolved from a single ancestor polypeptide after species divergence. An exception appears to be Lhcb3, the third component of LHCII trimers in seed plants, which does not form a group with Lhcb1-2 or Lhcbm and has subunit-specific features regardless of species. For example, as can be seen in the alignment shown in Fig. S6, two amino acid residues are conserved in Lhcb3 isoforms in different species but not in all other Lhcbm polypeptides. Lhcb3 orthologs are present in *P. patens* as well, suggesting its acquisition is associated with the evolution of land plants. This hypothesis is confirmed by the identification of several orthologs in non-seed plant EST databases (*Selaginella moellendorffii*, *Tortula ruralis*, *Marchantia polymorpha* and *Ceratopteris richardii*) while none was found in any algal species.

Lhcb4, Lhcb5 and Lhcb6 are generally classified as “minor” antennas [5]. Lhcb4 and Lhcb5 appear to be conserved during evolution and at least one ortholog could be identified in all the fully sequenced species as well as in many algae species. Lhcb4 appears to be more conserved than Lhcb5, where especially the loops connecting helix C to helix A and B differ in algae compared to plants (not shown).

Lhcb6 is only found in land plants, possibly suggesting that this protein plays a specific function related to the adaptation to a sub-aerial environment. To confirm this point, as done with Lhcb3, we searched in the public databases for sequences having significant similarity with Lhcb6 in algae and non-seed plants. No positive matches were found with algae, but sequences were retrieved in databases from several species of mosses, liverworts and ferns (*Selaginella lepidophylla*, *Tortula ruralis*, *Marchantia polymorpha* and *Adiantum capillus-veneris)*

Recently, the sequences of two additional putative PSII antenna subunits were identified and named respectively Lhcb7 and Lhcb8, the latter previously known as Lhcb4.3 [6]. A Lhcb7 ortholog was found in *Physcomitrella* which shows the Lhcb7-specific motif identified in *Arabidopsis*, poplar and rice, in agreement with the suggestion that it represents a distinct isoform [6]. In the case of Lhcb8, no ortholog was found in *Physcomitrella*, although this was expected since this polypeptide was only identified in dicots [6].

Finally, a new polypeptide was identified in *Physcomitrella* and named Lhcb9. Its position in the phylogenetic tree suggests high similarity with Lhcb proteins and supports its identification as a PSII antenna. A large number of EST clones were identified for Lhcb9 and the *PpLhcb9.1* isoform was represented by the highest number of clones among all the *Lhc* sequences identified, suggesting that the corresponding protein is probably abundant in *Physcomitrella* thylakoids.

### Lhc-like proteins in Physcomitrella are conserved with seed plants

Additional proteins sharing a common evolutionary origin with the Lhc proteins are present in Streptophyta. These polypeptides were previously given individual names but for the sake of a more systematic nomenclature they have been renamed as Lil (for Light harvesting like [5], [6]). In *Arabidopsis*, six different Lil polypeptides were identified and named Lil1-6, while a seventh Lhc-like protein is known as PsbS.

**Figure 3:**
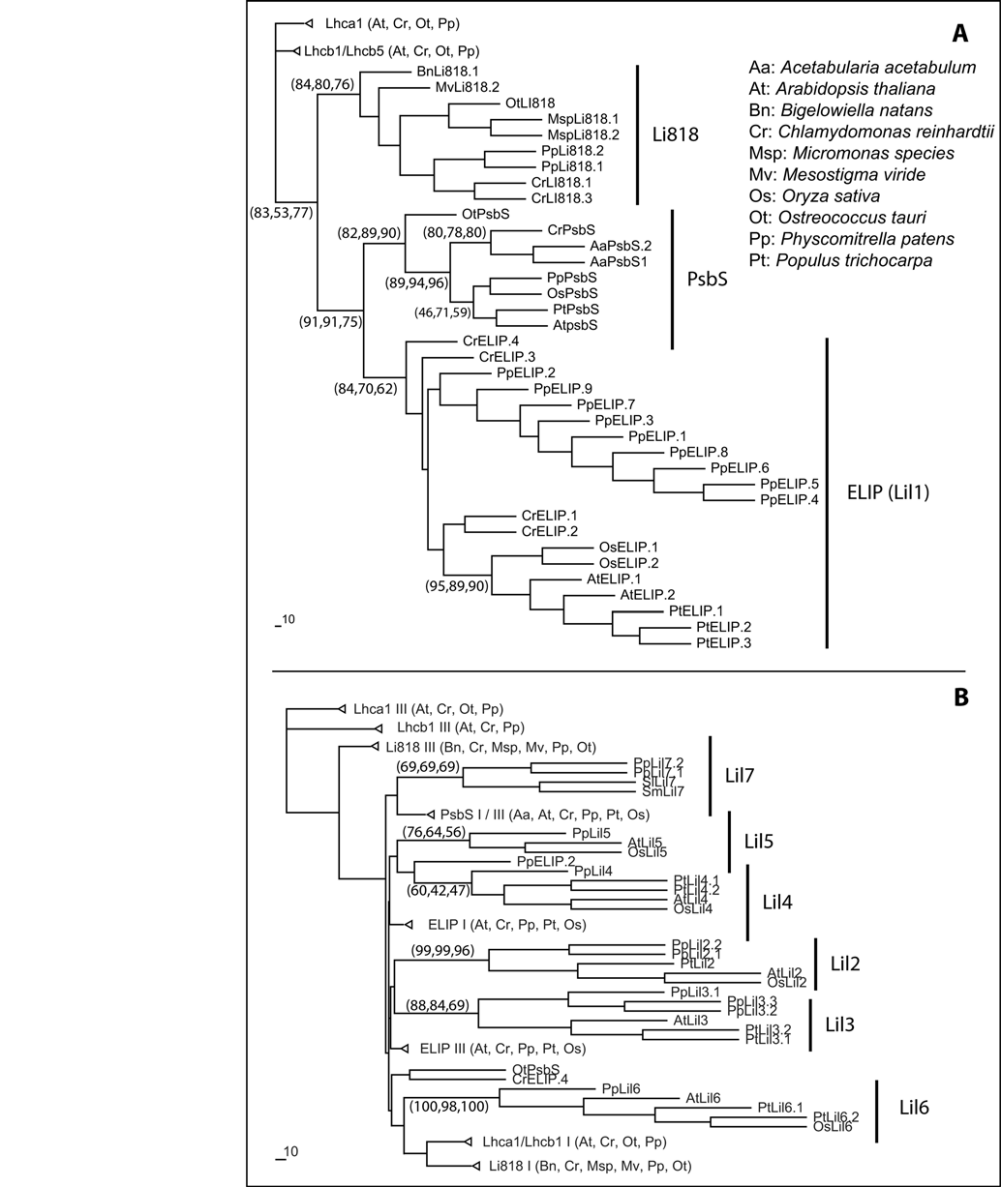
Phylogenetic tree of Lhc-like proteins. Phylogenetic tree of all Lhc-like proteins identified in *A. thaliana* (At), *Populus trichocarpa* (Pt), *O. sativa* (Os), *P. patens* (Pp), *C. reinhardtii* (Cr) and *O. tauri* (Ot). Sequences obtained from several algae species EST databases were also included (*Acetabularia acetabulum,* Aa, *Mesostigma viride,* Mv, *Micromonas sp.,* Msp, *Bigelowiella natans*, Bn). In Figure A only proteins with at least two homologous transmembrane helices are shown. In figure B all transmembrane helices were analyzed individually and roman numerals indicating the helix number. In both analyses some Lhca and Lhcb sequences were included as an external out-group. The tree shown was obtained with a maximum likelihood approach, but bootstrap values were obtained from maximum likelihood, NJD and maximum parsimony respectively. For clarity, bootstrap values are not shown when consistency was poor and when nodes are not useful to discriminate isoforms.

By searching the *Physcomitrella* expressed sequences databases, we found (list is reported in figure S5) a clear ortholog for PsbS, three Lil1 (better known as ELIPs) and Lil3 (OHP1) isoforms, and one isoform each for Lil2, Lil4, Lil5 and Lil6 (also known as HLIP, SEP1, SEP2 and OHP2). Interestingly, another Lhc-like gene, Li818, which was identified in several algae, is also expressed in *Physcomitrella* but to date was not found in seed plants [23]. A class of proteins with two predicted transmembrane helices, unique to *Physcomitrella*, was found among Lhc-like proteins. In order to remain consistent with our proposed nomenclature we named these polypeptides Lil7.1 and Lil7.2. The search for orthologs of this sequence in public databases only yielded results in two different club moss species, *Selaginella moellendorffii* and *Selaginella lepidophylla.* No other sequence with significant homology was found in seed plants or algae. Nevertheless, one transmembrane helix from PpLil7 has high homology with the first PsbS helix (61% identity and 88% similarity with the AtPsbS polypeptide). Genome sequence analysis confirmed the EST sequence data, with the significant exception that six additional ELIPs sequences were found, suggesting these isoforms are not expressed, at least under the growth conditions prior to mRNA isolation. To verify the reliability of these classifications all the sequences identified in *Physcomitrella* were then aligned with known sequences from *A. thaliana*, *O. sativa, Populus spp, C. reinhardtii*, *O. tauri,* the additional algal sequences and Lil7 from the two *Selaginella* species. These proteins diverge significantly and thus resulting phylogenetic trees must be considered with some caution. In order to obtain reliable results we only included the transmembrane helices, two when possible (Figure 3A) and one in the other cases (Figure 3B). Ortholog sequences did tend to consistently cluster together using different tree calculation methods. These analyses showed that all Lhc-like proteins identified in *A. thaliana* are also conserved in *P. patens* and the other seed plant species.

Finally, it is interesting to note that all Li818 sequences identified in several different algae species and *Physcomitrella*, are clear orthologs and show remarkable conservation.

### Biochemical characterization of the Physcomitrella photosynthetic apparatus

**Figure 4:**
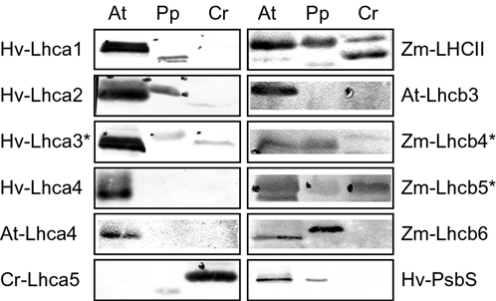
Identification of different Lhc proteins in *P. patens* by Western blotting. Thylakoids purified from *A. thaliana* (At), *P. patens* (Pp) and *C. reinhardtii* (Cr) were loaded on SDS PAGE and detected with antibodies raised against individual antenna polypeptides from *Arabidopsis* (At), *Hordeum vulgare* (Hv), *Zea mays* (Zm) and *C. reinhardtii* (Cr). 1 µg of Chls was loaded for each sample. When specific bands were detected the protein was identified from the apparent MW and these cases are indicated with an asterisk (*).

The identification of a sequence in the genome does not necessarily always correspond to the presence of the corresponding polypeptide: for example, the PsbS gene is present in the *Chlamydomonas reinhardtii* genome, but the corresponding protein has never been detected and in this organism NPQ is proposed to be PsbS independent [40]. Therefore, in order to complement the sequence analysis data we characterized the biochemical properties of antenna proteins in *P. patens*. We first analyzed thylakoid protein composition by Western blotting, using the two model species, *A. thaliana* and *C. reinhardtii,* as references. These models were chosen because of the large amount of data available concerning the biochemical properties of their photosynthetic apparatus (figure 4). However, the use of antibodies to compare different species has some limitations and the results should be treated with some caution. Two kinds of antibodies were produced by immunization with either a peptide or the whole protein. Since a greater number of potential epitopes is generated with a whole protein, we expected the subsequent antibodies to be less specific in distinguishing proteins from different species. In general, we observed that antibodies produced from peptides from seed plants (either *Arabidopsis* or barley) recognized *Arabidopsis* and *Physcomitrella* polypeptides, although with a lower apparent affinity for the latter. In one case, using the anti-Lhca3 antibody, we obtained a weak signal in *Chlamydomonas* as well and this result is consistent with the observed conservation of this polypeptide between different species. On the contrary, when antibodies raised against the whole Lhca3 protein were used, less specific signals were detected, e.g. Zm-LHCII or Cr-Lhca4.

Of note, the antibody against the seed plant Lhca4 (either from *Arabidopsis* or barley peptides) did not recognize a *Physcomitrella* protein, even when the assay was repeated using 10 times more *Physcomitrella* protein compared to *Arabidopsis,* suggesting that this isoform is not present in this moss.

**Figure 5:**
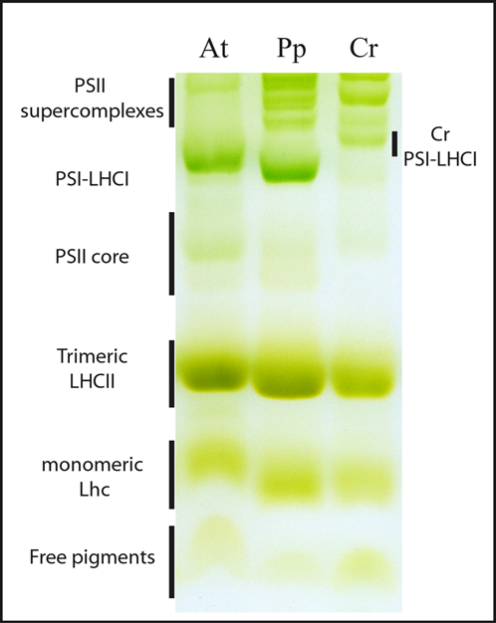
Comparison of thylakoid pigment binding complexes in *A. thaliana*, *P. patens* and *C. reinhardtii*. Non denaturing gel electrophoresis of pigment binding complexes from thylakoids from *A. thaliana* (At), *P. patens* (Pp) and *C. reinhardtii* (Cr) after solubilization with a final concentration of 0.8% α-DM. 30 µg of Chls were loaded for each sample. Different pigment binding complexes, identified from their mobility, are indicated on the left. The *C. reinhardtii* PSI-LHCI complex is shown on the right, because its mobility was different than in other species.

We then extended our analysis to the biochemical properties of moss thylakoids. *P. patens* protonema cells were harvested and thylakoid membranes purified. Native gel electrophoresis, after solubilization with a mild detergent, was used to analyze the composition of the pigment binding complexes. In figure 5, the electrophoretic profiles of thylakoids from *P. patens*, *Arabidopsis* and *Chlamydomonas* are compared. From their migration pattern different bands can be identified as (from the top): PSII-LHCII supercomplexes, PSI-LHCI, PSII core, trimeric and monomeric antennas and free pigments. Although the overall profiles are similar, differences can nevertheless be seen between species. In the case of *Physcomitrella*, the PSII core band is very faint and hardly detectable, while the PSII-LHCII supercomplexes are more abundant, especially with respect to *Arabidopsis*, suggesting a much stronger association between core and antenna polypeptides for PSII in this moss. To verify that increased stability of PSII-LHCII supercomplexes was not merely due to different lipid content/composition in the thylakoids membranes, we used stronger solubilization conditions but did not detect any appreciable differences. Thus high stability of PSII supercomplexes appears to be a peculiar trait of *P. patens*, and is similar to recent results found for *Inga sapindoides* [41]. This increased stability was also observed for *Chlamydomonas*, with the isolated PSII core also being poorly detected.

Another interesting difference is the apparent molecular weight of the PSI-LHCI complex: in seed plants, the PSI antenna system is known to be composed of four antenna polypeptides [42], [43], whereas in *Chlamydomonas* 9 to 11 proteins were found [44], [45]. The larger antenna of *Chlamydomonas* PSI-LHCI leads to an increased molecular weight of about 30%, which is consistent with its lower electrophoretic mobility compared to *Arabidopsis* (Fig. 5). In native PAGE the mobility of the *Physcomitrella* PSI-LHCI was similar or slightly higher than that of *Arabidopsis*, indicating that it is clearly smaller than in *Chlamydomonas*. The slightly higher mobility of the *Physcomitrella* complex may suggest that three LHCI subunits are present, which would be consistent with the absence of Lhca4 in the *Physcomitrella* genome. This larger mobility was consistently found in different repetitions, even when native gels were run following thylakoid solubilization with β-DM (*n*-dodecyl-β-D-maltoside), which leads to improved separation of PSI particles in native gels (not shown, [46]). Nevertheless, additional evidence is required to conclude that the *Physcomitrella* PSI antenna is indeed only composed of three polypeptides, which would also contradict the finding that LHCI polypeptides are organized as dimers [46].

### Physcomitrella responds to changes in light conditions

**Figure 6:**
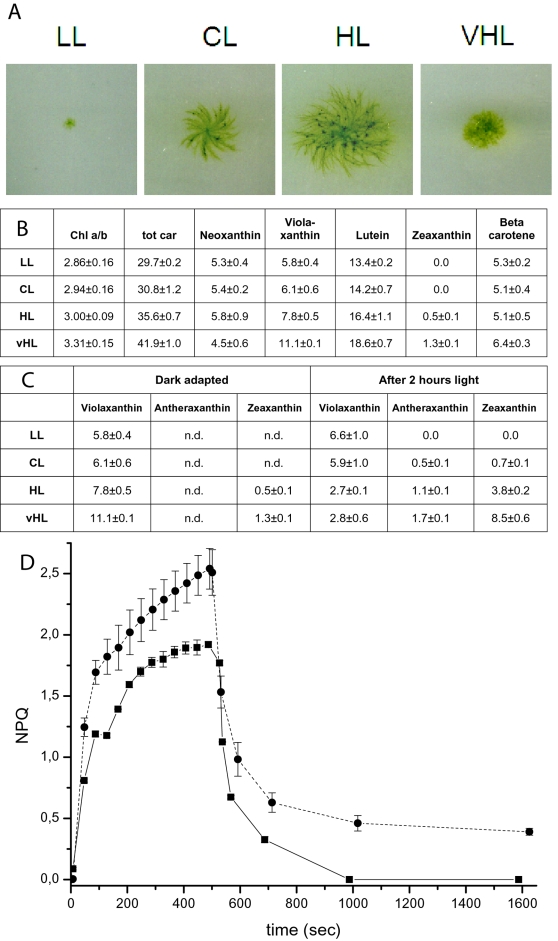
Acclimation, xanthophyll cycle and Non Photochemical Quenching in *P. patens*. WT plants were grown for 3–5 days under control conditions and then acclimated for 10 days to four different light intensities: Low Light 5 µE m^−2^ s^−1^ (LL), Control Light 20 µE m^−2^ s^−1^ (CL), High Light 70 µE m^−2^ s^−1^ (HL) and Very High Light 500 µE m^−2^ s^−1^ (VHL). A) Morphology of Acclimated plants. B) Pigment composition of acclimated plants after an overnight (14 h) dark adaptation. Carotenoid content is expressed as moles/100 moles of Chl a+b. C) Carotenoid composition comparison between dark adapted samples and samples treated for two hours with their corresponding growth conditions. D) Comparison of Non Photochemical Quenching (NPQ) kinetics after an overnight dark adaptation for *Physcomitrella* (grown in control conditions for 12 days, solid, squares) and *Arabidopsis* grown for 6 weeks at 100 µE m^−2^ s^−1^ (dashed, circles). Measurements were performed with saturating light at 4500 µE m^−2^ s^−1^ (duration time 600 µs) and actinic light at 1200 µE m^−2^ s^−1^ (which was on only for the first 8 minutes).

As mentioned above (see introduction), plant antenna proteins are involved in different photoprotection mechanisms, which belong to three categories depending on the activation time scale: NPQ, the xanthophyll cycle and long term acclimation. Since Lhc proteins are involved in all these mechanisms, as a first characterization of Lhc protein function in this organism, we tested photoprotection in *P. patens*. We grew *Physcomitrella* colonies under four different light intensities, ranging from 5 to 500 µE m^−2^ s^−1^ (Fig. 6A). We analyzed the pigment composition of these plants after an overnight dark adaptation: as shown in figure 6B, the Chl a/b ratio was different, suggesting that antenna size was modified in response to light treatment, similar to seed plants and green algae. We also observed that qP, which gives an estimation of the size of metabolic sinks, increased with increasing light growth intensity (not shown). These two parameters can generally be considered as indicating activation of an acclimative response in *Physcomitrella,* similar to previous observations in both green algae [47] and seed plants [15].

We also detected significant amounts of zeaxanthin in HL and VHL plants. Pigment binding properties were also analyzed by HPLC after two hours of light treatment: as shown in figure 6C, after this short light treatment we observed an increase in the zeaxanthin and antheraxanthin content with a corresponding decrease in violaxanthin, implying the presence of an active xanthophyll cycle in this moss.

Finally, we analyzed the presence of NPQ in *Physcomitrella*. This experiment was of particular interest, because while acclimation and xanthophyll cycle are known to be present in green algae as well, it was recently suggested that the amplitude and mechanisms for NPQ are significantly different in seed plants vs. green algae [40]. In figure 6D, NPQ kinetics measured with a PAM fluorometer are shown, and compared to *Arabidopsis*. The results clearly showed that a strong NPQ is induced in *Physcomitrella* and that this quenching might be even faster than in seed plants, as shown by its very rapid relaxation after the light is switched off. Although a direct correlation is not possible, the presence of an active NPQ is consistent with the observed presence of an expressed PsbS protein in *Physcomitrella* (Fig. 4). Antibody signal was weaker than in *Arabidopsis,* although this may be due only to a different antibody affinity for polypeptides from different species.

## Discussion

**Figure 7:**
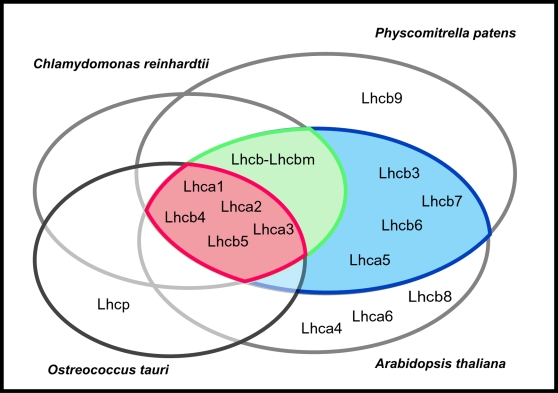
Distribution of Lhc subunits in different green lineage organisms. Lhca/b polypeptides were grouped according to the presence of a genuine homologue in the different fully sequenced organisms included in this study. *Arabidopsis thaliana* is the only seed plant shown because no differences were detected with poplar and rice a part from Lhcb8 which is absent in the latter. Lhca4 is indicated with an asterisk because, although absent in *P.patens* it was identified in other non seed plant species. The different colors indicate: i) Proteins commonly found in all organisms (red), ii) Proteins found in all organisms except *O. tauri* (blue), and iii) Proteins found only in land plants (green).

In this work we used complementary approaches to characterize the antenna system of the moss *Physcomitrella patens.* The comparison of antenna polypeptide sequences with proteins from other organisms, either seed plants or green algae, showed that not all different isoforms are found in all the organisms examined. *P. patens* diverged from seed plants shortly after land colonization: thus, all proteins which are present both in *Physcomitrella* and seed plants but not in algae must have evolved very early after land colonization. These proteins are interesting because they might be involved in the adaptation to this new environment and its challenges for photosynthetic organisms such as the presence of direct illumination, low CO_2_ availability and fast O_2_ diffusion. As shown in figure 7, four Lhc polypeptides were found to be specific to land plants. Among these, Lhcb3 and Lhcb6 appear to be the most remarkable since they are highly expressed in both seed plants and *Physcomitrella*.

### A “functional core” antenna system is conserved within the green lineage

Five Lhc polypeptides were always found in all the fully sequenced organisms considered here: Lhca1, Lhca2, Lhca3, Lhcb4, Lhcb5 (Fig. 1, 2 and 7). Their presence in several different organisms suggests that these antenna polypeptides originated early during the differentiation of green lineage organisms, before the divergence of Streptophyta from Chlorophyta. Among these, Lhca3 and Lhcb4 appear to be the most conserved components for PSI and PSII, respectively, which is in agreement with a recent phylogenetic analysis of EST sequences in algae and seed plants [32]. Orthologs of all these polypeptides were also identified in *Mesostigma viride*, which represents the deepest branch of Strephophytae [35], [48], and is thus consistent with the early evolution hypothesis.

It is interesting to observe that although the expression of antenna subunits is known to vary with different light conditions, in *Arabidopsis* plants acclimated to different light conditions the content of the five conserved polypeptides (Lhca1, Lhca2, Lhca3 and Lhcb4 and Lhcb5) with respect to reaction centre did not change [49]. Thus, these proteins are not only present in most green lineage organisms but, at least in seed plants, they are always present regardless of environmental conditions. These two considerations, taken together, suggest that they represent the “functional core” of the Photosystem I and II antenna system in green lineage organisms. The relevance of the presence of such a stable “core antenna system” can be exemplified by considering the phenotype of Chl b-less mutants, where antenna proteins are largely depleted: these plants not only harvest light less efficiently but are also highly sensitive to light stress [50]. Thus, the presence of such a constitutive antenna system in all organisms was probably maintained during evolution because it assures the level of light harvesting and photoprotection necessary in any terrestrial or aquatic environment, with limiting or excess light conditions.

On the contrary, additional antenna subunits such as Lhcb3 and Lhcb6 are differently distributed in different organisms suggesting they may play a role in more specific environments. Accordingly, in *Arabidopsis thaliana* the content of both these polypeptides was shown to respond to growth conditions suggesting an advantage in some conditions [49].

### Distribution of red forms in PSI antenna polypeptides

**Figure 8:**
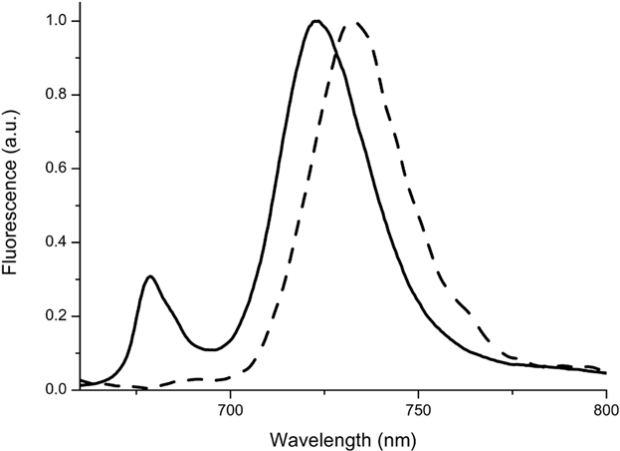
Low energy absorption forms in Photosystem I from *P. patens*. Low Temperature (77 K) fluorescence of PSI-LHCI purified from *Physcomitrella* (solid) and *Arabidopsis* (dashed) are shown. All spectra were normalized to the maximum value.

As shown in figure 7, one PSI antenna protein, Lhca4, is absent in *Physcomitrella* but present in seed plants. This result was also confirmed by Western blotting: as shown in figure 4, while we were able to detect all other Lhc isoforms in *Physcomitrella*, we could not detect Lhca4 even by loading 10 times more sample. The absence of a Lhca4 ortholog in *Physcomitrella patens* is also confirmed by the LT fluorescence measurements reported in Figure 8: in fact, PSI-LHCI purified from *Physcomitrella* peaks at 725 nm, and thus is shifted towards blue compared to AtLhca4, whose emission was identified at 735 nm [51], similar to PSI-LHCI purified from *Arabidopsis* (Fig. 8). This confirms that a genuine functional AtLhca4 ortholog, reproducing its fluorescence properties, is indeed absent in *Physcomitrella patens*.

To investigate the distribution of the Lhca4 protein in more detail, we searched the public databases for sequences similar to AtLhca4, and specifically examined whether the similarity was correlated with the presence of an asparagine residue as a ligand for Chl A5, which was shown to be essential for the presence of red-most Chl forms, a distinct property of AtLhca4 [51]. Sequences with the highest level of conservation belong to seed plants and all contain N as the ligand for Chl A5, both in monocots and dicots. Among non seed plants, a sequence identifiable as Lhca4 was found only in the lycophyte *Selaginella moellendorffii* and the liverwort *Marchantia polymorpha.* When the search was limited to algae, the retrieved sequences not only showed a lower level of similarity but also a histidine residue as the Chl A5 ligand. Examples of Lhc proteins possessing an asparagine as the ligand for Chl A5 is in *Chlamydomonas*, but the primary sequence shows an overall low level of sequence conservation with AtLhca4 [36]. Consistently, the *Chlamydomonas* PSI antenna has a smaller red shift and fluorescence emission peaks around 715 nm [52]. Thus, although homologous sequences for Lhca proteins are present in both Chlorophyta and Streptophyta, the specific combination responsible for the strong red shift in *Arabidopsis* Lhca4 evolved only in plants early after land colonization.

In *Arabidopsis*, besides Lhca4, red-shifted forms (emitting at 725 nm) are also associated with Lhca3, which carries an asparagine residue as a ligand for Chl A5 [51]. Lhca3 orthologs with the conserved asparagine Chl A5 ligand are present in *Physcomitrella*. *In vitro* reconstitution experiments with PpLhca3.1-2 demonstrated these proteins have LT fluorescence at 725 nm, which is the same as for AtLhca3 (not shown). Thus, this subunit is most likely responsible for red-most emission in *Physcomitrella* cells.

Interestingly, while Lhca3 is clearly conserved in all members of the green lineage, Chl A5 ligand is not conserved and asparagine is not found in any algae species. This is the case of *Chlamydomonas* and *Ostreococcus* orthologs which have an histidine as the Chl A5 ligand and the PSI antenna consistently shows a smaller red shift (*C. reinhardtii*, [52]) or is missing red emitting forms (*O. tauri*, [30]). In *Mesostigma viride,* which is a Streptophyta as well as plants, the Lhca3 sequence contains a histidine as the Chl A5 ligand and thus smaller red-shifted forms are likely. On the contrary, in all plant species analyzed, included non-seed species (*Marchantia polymorpha*, *Selaginella moellendorffii*, *Ceratopteris richardii*) all Lhca3 sequences contained an asparagine as the Chl A5 ligand suggesting red-shifted emission. These results suggest that during the evolution of the Streptophyta towards seed plants there was a progressive increase in the amplitude of red forms relative to bulk Chl absorption and a wavelength shift towards lower energy. A possible explanation is that absorption at longer wavelengths provided a significant advantage in the terrestrial environment. In fact, because long wavelengths are preferentially absorbed by water, red Chl forms may not provide a significant contribution to photosynthesis. By contrast, these wavelengths constitute a significant part of the light spectrum in the aerial environment, especially under shade conditions within a canopy [53]. An additional/alternative explanation is that these chlorophylls could contribute to photoprotection from light excess. In fact, the pronounced red Chl forms funnel singlet excited states to sites where any Chl triplet that is generated can be effectively quenched by nearby carotenoids [54], [55]. Thus, the presence of Chl molecules with low energy levels would help plants resist the increased photo-oxidative stress experienced in sub-aerial environments.

The presence of such red-shifted Chls in the PSI-LHCI supercomplex of land organisms also has consequences on energy distribution: at room temperature approximately 90% of excited states are localized in red forms and then transferred to the reaction center [56]. This peculiar energy distribution does not have effects on the overall photochemical efficiency because PSI is very efficient in charge separation and PSII, which is slower, is excitonically separated from PSI being segregated in grana [57], [58]. It is interesting to mention that the thylakoid organization with grana stacking was shown to be generally absent in most algal species. In fact, although some thylakoid appression is found in many algae, an organization as higher plants grana was found only in later taxa of the Streptophyta line (Coleochaetales and Charales) [59], [60]. It thus tempting to hypothesize that these differences are somehow correlated: evolution of grana stacking may have allowed the presence of strong red-shifted forms in Photosystem I, without causing spillover and reduction of PSII photochemical efficiency.

### The PSII antenna system in photosynthetic eukaryotes: adaptations to the aerial environment

The major PSII antenna in seed plants is composed of the Lhcb1, Lhcb2 and Lhcb3 proteins. Lhcb1 and Lhcb2 are highly similar and are present in multiple copies (5 and 4 respectively in *Arabidopsis*, [5]), whereas Lhcb3 is more divergent, is encoded by a single copy gene and was shown to have distinct biochemical and spectroscopic properties [39]. Homologous proteins are present in many other plants and algae species (called Lhcbm), except for *Ostreoccoccus* where no ortholog was found and the major antenna complex is composed of Lhcp polypeptides, typical of prasinophytes [30]. Despite their absence in *O. tauri*, however, Lhcbm-like polypeptides were found in all other species analyzed from both Streptophyta and Chlorophyta: it is thus highly likely that these subunits appeared very early during green algae evolution, before the divergence of these two phyla.

Similarly to seed plants, in *Physcomitrella* and all other algae species analyzed in this study, major LHCII components are present in multiple copies. Nevertheless, these genes are more similar to each other than to proteins from other organisms (Fig. 2). Our sequence comparison (e.g. Helix B domain alignment shown in Figure S6) clearly highlighted only a few amino acid substitutions which appear to be species-specific. On this basis we conclude that the genes encoding the major LHCII trimeric complex differentiated after species divergence. This strong independent tendency among all the organisms examined for multiple genes encoding multiple LHCII isoforms, suggests the action of a selection pressure. This selection pressure was also present in *Ostreococcus* which also has multiple copies of the genes encoding its major antenna. One possible explanation is that different isoforms play specialized roles under different environmental conditions. However, the sequence singularities of each isoform are generally very small and, although the possibility could not be excluded, it seems unlikely that these could confer an individual specialized function on each polypeptide. Furthermore, the *in vitro* reconstitution of different barley Lhcb1 isoforms showed that, at least in the monomeric form, their spectroscopic and pigment binding properties were essentially identical [61].

An alternative explanation could be the need for high expression levels of the LHCII antenna complex. Considering that this major antenna complex is very abundant and plays a fundamental role both in light harvesting and photoprotection [62], its expression level probably responds to contrasting stimuli. For example, it has been shown that the LHCII polypeptide content is reduced in high light conditions [49], but also that at least one trimer per PSII core is always maintained even in extreme light stress [63]. A single copy gene, with a unique promoter and a relatively small number of regulatory regions, may not sustain this massive and regulated protein expression, whereas multiple copies may achieve an optimal integrated regulation of the LHCII content. The observation that different Lhcb1 isoforms, although very similar, are distinctly regulated according to environmental conditions [64] fits with this hypothesis.

Lhcb3 shows a different pattern from the other LHCII polypeptides: sequence differences are subunit-specific rather than species-specific. Indeed, biochemical studies in barley and *Arabidopsis* showed that Lhcb3 has properties which are distinct from those of Lhcb1-2 [39], [65]. A Lhcb3 ortholog is present in seed plants [6], *Physcomitrella* and other non-seed plants, but not in any green algae analyzed so far [32], suggesting a specialized role for this polypeptide within LHCII, possibly related to life in land environments.

Lhcb6 also appeared during the evolution of land plants and no ortholog is found in algae, either in EST or genomic databases [32]. Lhcb6 was proposed to play a structural role in PSII supercomplexes: in plants depleted of this polypeptide, the PSII core association of a subpopulation of LHCII trimers, the M-trimers, was destabilized [63], [66]. Consistent with this hypothesis, both Lhcb6 polypeptides and M-trimers are found in the liverwort *Marchantia polymorpha* but not in algae PSII supercomplexes [58], [67]. Considering that Lhcb3 is enriched in M-trimers, it is tempting to speculate that the evolution of these two proteins in land plants allowed the differentiation of different trimeric LHCII complexes within PSII supercomplexes.

In addition to this structural role, Lhcb6 may be involved in the regulation of light harvesting. *Arabidopsis* mutants depleted in Lhcb6 showed a strong reduction in NPQ [66]. Thus the presence of Lhcb6 (and possibly Lhcb3) may be correlated with the presence of a fully active NPQ in *P. patens* and seed plants, in a different scenario from green algae where Lhcb6 is absent and NPQ works via a different mechanism [40]. Following this sequence of events, the evolution of Lhcb6 (and Lhcb3) in organisms emerging from aquatic environments led to improved adaptation to the aerial environment by leading to NPQ in the form now found in seed plants. In conclusion, although not all mechanisms are fully understood, the hypothesis that, besides PsbS, Lhcb6 and possibly Lhcb3 play a relevant role in regulating PSII antenna function is worth verifying by future research.

Finally, other Lhcb polypeptides appeared only at later stages in the evolution of the green lineage: Lhcb7, Lhcb8 and Lhcb9. Lhcb7 is specific for land plants, whereas Lhcb8 is typical of dicots only. Lhcb9, on the contrary, is only found in *Physcomitrella* and no ortholog was found in any other organism, including non- seed plants. This peculiar distribution suggests that the function of this protein is limited. Its high expression level, however, as judged from the number of EST clones, leaves open the possibility that it may play a specific role in the *Physcomitrella* growth environment. For example, sunflecks are particularly dangerous for deep-shade plants because the sudden transition from low to high light drives triplet and reactive species formation: Lhcb9 could play a protecting role in these particular conditions, although other scenarios are also possible.

### Phylogeny of Lhc-like sequences: evolution of photoprotection mechanisms

In *Physcomitrella* sequence databases we also identified several Lhc-like proteins, which show far less conservation than Lhc complexes [1], [68]. We identified at least one isoform of PsbS and ELIPs in all the fully sequenced organisms analyzed. The remaining Lil2-6 proteins identified in *Arabidopsis,* poplar and rice [6] were all conserved in *P. patens* suggesting that their physiological function is maintained among land plants.

Finally, we found that the Li818 protein was also conserved during evolution in algae and *Physcomitrella* [69]. As shown in figure 3, its level of sequence similarity with other Lil proteins is remarkably high, suggesting a conserved function. In algae this protein was shown to be preferentially expressed in high light conditions [69] and hence it may play a role in photoprotection. Very recently, preliminary results suggest that Li818 is the protein responsible for NPQ in *C. reinhardtii* [70]. If this is confirmed in other algae species, then *P. patens* would be the only organism known to date to have both subunits responsible for NPQ in plants and algae, PsbS and Li818 respectively. Further studies, however, are necessary to clarify their function in *P. patens*.

## Materials and Methods

### Sequence retrieval

Sequence searches were performed in PHYSCObase database (http://moss.nibb.ac.jp/blast/blast.html) that contained 196,310 sequences from clones isolated from three *Physcomitrella patens* full-length-enriched cDNA libraries [23], sequenced both from the 5′ and 3′ ends and then assembled in 27.546 contigs. The database was initially queried for Lhc superfamily members by conducting tBLASTn searches using the *Arabidopsis* and *Chlamydomonas* proteins as queries [5], [30], [36]. Each contig which showed some degree of homology with an Lhc family member was then analysed individually to eliminate artifactual sequences (duplicate contigs, incomplete contigs, badly assembled contigs showing only partial homology with *Lhc* sequences). The *contig1521* and *contig9976* (proteins were named PpLhca3.2 and PpLhca3.4) were incomplete in the database and were completed by analysing the single cDNA clones used for assembling the sequence. The number and quality of clones used for assembling each contig was also checked. Most of the contigs were assembled from a number of high quality sequences representative of the ORF sequence (criterion: at least 5 clones, sequenced both from the 5′ end and 3′ end, overlapping and identical). This was the case of most Lhc antenna proteins where dozens of clones were retrieved for each gene. In the case of a few other polypeptides, contigs were assembled from a smaller number of clones: *contig14942* (PpELIP.1) was assembled from only one clone sequenced in both directions; *contig8165* (PpLil7.2), *contig11093* (PpLil3.1), *contig11590* (PpLil7.1), *contig133* (PpELIP.2) and *contig7808* (PpLil5) were assembled from 2, 2, 4, 3 and 3 clones respectively although the sequence quality was always high. *Contig7368* (PpLil4) apparently lacked a few amino acids at the N-term while PpLhcbm13, PpELIP.3, PpLhcb7, PpLil3.2 and PpLil3.3 do not have a contig sequence and the respective sequences were found by cDNA analysis. The information on the genome sequence (http://genome.jgi-psf.org//Phypa1_1/Phypa1_1.home.html) was used to check the completeness and reliability of the contig sequences. All contig sequences were confirmed by the genome sequence except PpELIP.3 which was shown to be missing a few amino acids at the N terminus. Genome analysis also led to the identification of additional proteins, ppLhca5, PpHLIP.2 and PpELIP.4-9. When possible, sequence nomenclature was based on homology to *Arabidopsis* proteins and numbered according to the number of clones used for assembling the contig, assuming that this number was approximately correlated with gene expression. For analyses in other organisms, tBLASTn searches were launched both at NCBI (http://www.ncbi.nlm.nih.gov/BLAST/) and TIGR (http://plantta.tigr.org/) databases.

### Sequence analysis

Sequence alignments were generated using clustalW and manually corrected using BioEdit. The residues close to the three transmembrane helices were considered in the analysis, to include a total region spanning 165 amino acid residues as previously described (Fig. S2, [3], [30]). In the case of Lhc-like sequences only transmembrane regions were retained and alignments contained 56 and 28 residues respectively for figure 3A and B. Protein sequences (Fig. S2) instead of DNA were analyzed because the third codon tends to be random and the protein sequence is less sensitive to biased G+C content, as is the case of the *Chlamydomonas* genome. Phylogenetic trees were generated using parsimony, Neighbor-Joining Distance (NJD) and maximum likelihood approaches in the Phylogenetic Inference Package (PHYLIP) version 3.67 (http://evolution.genetics.washington.edu/phylip.html). Specifically, the PROTPARS function was used for parsimony analyses and the sequence input order was randomized (10 jumbles). The PROTDIST application was used to create distance matrices with the JJD model for amino acid substitution and the coefficient of variation of substitution rates among positions. Neighbor-Joining Distance (NJD) trees were generated using the NEIGHBOR program and consensus trees were generated with CONSENSE. The PHYML program [71], [72] was used for the maximum likelihood analyses (http://atgc.lirmm.fr/phyml/) utilizing the WAG amino acid substitution matrix, with gamma correction (4 categories) and accounting for the number of invariant sites. For all analyses, trees confidence values were obtained by bootstrapping 100 data sets (SEQBOOT).

### Physcomitrella growth and thylakoids isolation

Protonemal tissue of *P. patens*, Gransden wild-type strain was grown on PpNO3 minimum medium [73] solidified with 0.7% Purified Agar (Euromedex, Mundolsheim CEDEX). Plants were propagated under sterile conditions on minimum medium in 9 cm Petri dishes overlaid with a cellophane disk (Cannings, Bristol) as described by [74]. Plates containing plant samples were placed in a growth chamber under controlled conditions: 22°C day/21°C night temperature, 16 h light/8 h dark photoperiod and a light intensity of 20 µE m^−2^ s^−1^ (control conditions). Thylakoids from two weeks old plants (protonemal tissue) were prepared following the same protocol used for seed plants with minor modifications [75]. Tissues were harvested and freshly homogenized in cold extraction buffer (0.5% milk powder, 0.4 M NaCl, 20 mM Tricine-KOH pH 7.8 and 1mM ε-aminocaproic acid). After filtration, samples were precipitated by centrifugation at 1500 g for 15 min at 4°C and then resuspended in an ipotonic buffer (15 mM NaCl, 5 mM MgCl_2_ and 20 mM Tricine-KOH pH 7.8). After centrifugation for 15 min at 10000g at 4°C, thylakoids were resuspended in a buffer containing 50 % Glycerol, 15 mM NaCl, 5 mM MgCl_2_ and 10 mM HEPES-KOH pH 7.5. Thylakoids were frozen in liquid nitrogen and stored at –80°C until use.

### Biochemical analyses

Non-denaturing Deriphat-PAGE was performed following the method described in [76] with the modification reported in [46]. Thylakoids with a at Chl concentration of 1 mg/ml were solubilized with an equal volume of 1.2% or 2% of α and β-DM respectively (Anatrace©, Maumee, OH). Thus, final concentrations of Chls, α and β-DM were 0.5 mg/ml, 0.6 and 1%, respectively. Solubilized thylakoids were vortexed for 1 min, left on ice for 10 min and then centrifuged at 10.000*g* for 15 min to pellet unsolubilized material. 30 µg of chlorophyll was loaded per each gel lane. Purification of pigment binding proteins from thylakoids was performed by sucrose gradient ultracentrifugation upon solubilization of membranes with a final concentration of 0.8 % α-DM as described in [77].

### SDS PAGE Electrophoresis and Western blotting analysis

SDS-PAGE analyses were performed as in [43]. After SDS-PAGE, proteins were transferred onto a nitro-cellulose membrane (Sartorious AG, Gottingen Germany) using a blot system from Biorad (Hercules, CA) and detected with specific antibodies.

### Spectroscopy and pigment analysis

Absorption spectra were recorded using a Cary 300 (Varian Inc.) spectrophotometer, in 10 mM HEPES pH 7.5, 0.2 M sucrose and 0.06% α-DM. Low Temperature fluorescence emission spectra were measured using a Cary Eclipse (Varian Inc.) and corrected for instrument responses. Samples were excited at 440, 475 and 500 nm. The spectral bandwidth was 5 nm (excitation) and 3 nm (emission). The chlorophyll concentration was approximately 0.02 µg/ml in 60% glycerol, 10 mM HEPES and 0.03% α-DM. The chlorophyll to carotenoid ratio and Chl a/b ratio were independently measured by fitting the spectrum of acetone extracts with the spectra of individual purified pigments [78] and by HPLC analysis as described in [79].

### In vivo chlorophyll fluorescence measurements

Chlorophyll fluorescence was measured at room temperature with a PAM-101 fluorometer with a saturating light at 4500 µE m^−2^ s^−1^ and actinic light of 1200 µE m^−2^ s^−1^. Before measurements, plates were dark-adapted overnight at room temperature. The parameters Fv/Fm, NPQ and qP were calculated respectively as (Fm-Fo)/Fo, (Fm-Fm')/Fm' and (Fm'-F)/(Fm'-Fo) [80].

## Supporting Information

Figure S1 Physcomitrella patens Lhc polypeptides identified in Physcobase. When clear homology was seen the name was derived from the A. thaliana ortholog. This nomenclature was not used for components of the major LHCII, named Lhcbm1-13 and Lhcb9 which is specific to P. patens. Contig and scaffold number and the best hit with the tblastn search at NCBI (http://www.ncbi.nlm.nih.gov/BLAST/) are also reported. In a few cases the contig sequence was missing in the database and it was reconstructed by analysis of individual EST clones. Lhca5 was not found in the EST database and was retrieved after genome analysis.
(0.07 MB DOC)

Figure S2 Example of sequence alignments used for generating phylogenetic trees. Only two genes (Lhcb1.1 and Lhca1 from A. thaliana) are shown for clarity. Only the most conserved regions which are indicated in gray were considered for the analysis, as in (Dunford et al., 1999).
(8.21 MB TIF)

Figure S3 List of all polypeptide sequences included in the analysis.
(0.11 MB DOC)

Figure S4 Phylogenetic tree of Lhca antenna proteins. A Phylogenetic tree was generated as described for figure 1. In this case, the number of PSI antenna sequences was increased. A total of 100 sequences were analyzed. Four Li818 sequences from P. patens (Pp), C. reinhardtii (Cr) and O. tauri (Ot) where also included as an external out-group. The tree shown was built using a maximum likelihood approach, but bootstrap values were obtained from maximum likehood, NJD and maximum parsimony approaches, respectively. For clarity, these values are not shown when consistency was poor or when node bootstrap values were not sufficiently significant to discriminate isoforms.
(7.64 MB TIF)

Figure S5 P. patens Lhc-like polypeptides identified in Physcobase. When clear homology was seen the name was derived from the A. thaliana ortholog. In the case of Li818, names were derived from Chlamydomonas nomenclature, since no homologs were found in seed plants. Contig number and the best hit with the tblastn search at NCBI (http://www.ncbi.nlm.nih.gov/BLAST/) are also reported. In a few cases the contig sequence was missing in the database and it was reconstructed by analysis of individual EST clones. Several Lil1 and one Lil2 isoform were not found in the EST database and were retrieved after genome analysis.
(0.05 MB DOC)

Figure S6 Alignment of PSII major antenna complex polypeptides from C. reinhardtii, P. patens and A. thaliana. The region corresponding to the first transmembrane helix (also called Helix B) is shown. Residues showing species dependent differences are indicated with an asterisk (*).
(13.94 MB TIF)
