# Supplementary material for: In Silico and Biochemical Analysis of Physcomitrella patens Photosynthetic Antenna: Identification of Subunits which Evolved upon Land Adaptation
Source: PLoS One. 2008 Apr 30;3(4):e2033. doi: 10.1371/journal.pone.0002033 (PMC2323573; doi:10.1371/journal.pone.0002033)
Supplement: Figure S1 — Physcomitrella patens Lhc polypeptides identified in Physcobase. When clear homology was seen the name was derived from the A. thaliana ortholog. This nomenclature was not used for components of the major LHCII, named Lhcbm1-13 and Lhcb9 which is specific to P. patens. Contig and scaffold number and the best hit with the tblastn search at NCBI (http://www.ncbi.nlm.nih.gov/BLAST/) are also reported. In a few cases the contig sequence was missing in the database and it was reconstructed by analysis of individual EST clones. Lhca5 was not found in the EST database and was retrieved after genome analysis. (0.07 MB DOC) [file pone.0002033.s001.doc]

|  | | | **Best Hit** | | |
| --- | --- | --- | --- | --- | --- |
| **Name** | **Contig** | **Scaffold** | **Organism** | **Annotation** | **E value** |
| PpLhca1.1 | *Contig7360* | 158:530336-532224 | *A. thaliana* | chlorophyll binding (LHCA1) | 4E-83 |
| PpLhca1.2 | *Contig11379* | 161:214220-216144 | *A. thaliana* | chlorophyll binding (LHCA1) | 5E-90 |
| PpLhca1.3 | *Contig9113* | 247:100794-102610 | *Zea mays* | clone 13119 mRNA sequence | 4E-92 |
| PpLhca2.1 | *Contig2691* | 32:21187-22202 | *Pinus sylvestris* | mRNA for type II chlorophyll a/b binding protein of LHCI | 5E-98 |
| PpLhca2.2 | *Contig1907* | 330:317734-370943 | *O. sativa* | cDNA clone:OSIGCSA039A18 | 9E-98 |
| PpLhca2.3 | *Contig2379* | 241:342278-343566 | *O. sativa* | cDNA clone:OSIGCSA039A18 | 6E-102 |
| PpLhca2.4 | *Contig14811* | 651:2617-4370 | *Pinus sylvestris* | mRNA for type II chlorophyll a/b binding protein of LHCI | 5E-104 |
| PpLhca3.1 | *Contig1522* | 214:742990-744999 | *Pinus sylvestris* | mRNA for type III chlorophyll a/b binding protein LHCI | 9E-99 |
| PpLhca3.2 | *Contig1521* | 429:221543-222804 | *Pinus sylvestris* | mRNA for type III chlorophyll a/b binding protein LHCI | 9E-88 |
| PpLhca3.3 | *Contig7029* | 197:593587-594900 | *Pinus sylvestris* | mRNA for type III chlorophyll a/b binding protein LHCI | 8E-92 |
| PpLhca3.4 | *Contig9976* | 214:743641-744934 | *Pinus sylvestris* | mRNA for type III chlorophyll a/b binding protein LHCI | 1E-88 |
| PpLhcbm1 | *Contig959* | 19:147444-148757 | *Panax ginseng* | cab mRNA for chlorophyll a/b binding protein | 5E-108 |
| PpLhcbm2 | *Contig11084* | 27:773051-774378 | *Panax ginseng* | cab mRNA for chlorophyll a/b binding protein | 9E-112 |
| PpLhcbm3 | *Contig11400* | 177:82509-83748 | *Ginkgo biloba* | nuclear-encoded chloroplast chlorophyll a/b binding protein mRNA | 7E-110 |
| PpLhcbm4 | *Contig11353* | 182:654564-655721 | *Pinus palustris* | type 2 light-harvesting chlorophyll a/b-binding polypeptide (Lhcb2) | 2E-122 |
| PpLhcbm5 | *Contig10369* | 52:1408943-1410314 | *Panax ginseng* | cab mRNA for chlorophyll a/b binding protein | 6E-122 |
| PpLhcbm6 | *Contig11007* | 256:108568-109709 | *Amaranthus hypochondriacus* | Lhcb2*Ah1 | 5E-121 |
| PpLhcbm7 | *Contig14956* | 186:230061-231245 | *Pinus palustris* | type 2 light-harvesting chlorophyll a/b-binding polypeptide (Lhcb2) | 6E-120 |
| PpLhcbm8 | *Contig10657* | 1:3703434-3704795 | *Pinus palustris* | type 2 light-harvesting chlorophyll a/b-binding polypeptide (Lhcb2) | 2E-119 |
| PpLhcbm9 | *Contig11056* | 76:1455892-1457134 | *Vigna radiata* | LHCII type I chlorophyll a/b-binding protein (CipLhcb1*3) | 6E-120 |
| PpLhcbm10 | *Contig9567* | 12:2735929-2737299 | *Pinus palustris* | type 2 light-harvesting chlorophyll a/b-binding polypeptide (Lhcb2) | 9E-122 |
| PpLhcbm11 | *Contig14895* | 463:126335-127805 | *Ginkgo biloba* | nuclear-encoded chloroplast chlorophyll a/b binding protein mRNA | 4E-122 |
| PpLhcbm12 | *Contig7430* | 51:1790574-1843613 | *Pinus palustris* | type 2 light-harvesting chlorophyll a/b-binding polypeptide (Lhcb2) | 8E-120 |
| PpLhcbm13 |  | 13:1336588-1337813 | *Panax ginseng* | cab mRNA for chlorophyll a/b binding protein | 2E-115 |
| PpLhcb3.1 | *Contig10429* | 254:22926-24576 | *O. sativa* | cDNA clone:001-013-H12 | 3E-110 |
| PpLhcb4.1 | *Contig1795* | 472:146582-148016 | *Vigna radiata* | chlorophyll a/b binding protein CP29 (CipCp29) | 6E-93 |
| PpLhcb4.2 | *Contig14800* | 155:281926-283416 | *Vigna radiata* | chlorophyll a/b binding protein CP29 (CipCp29) | 1E-90 |
| PpLhcb5.1 | *Contig690* | 628:9551-19231 | *Pinus sylvestris* | Lhcb5*1 mRNA encoding Lhcb5 protein | 7E-100 |
| PpLhcb5.2 | *Contig6913* | 628:9534-19424 | *Pinus sylvestris* | Lhcb5*1 mRNA encoding Lhcb5 protein | 3E-99 |
| PpLhcb6.1 | *Contig2038* | 28:2045733-2046918 | *Nicotiana tabacum* | chloroplast pigment-binding protein CP24 (Lhcb6) | 1E-105 |
| PpLhcb6.2 | *Contig2039* | 2016702-2018040 | *Nicotiana tabacum* | chloroplast pigment-binding protein CP24 (Lhcb6) | 1E-105 |
| PpLhcb7 |  | 215:877716-880966 | *O. sativa* | Os09g0296800 (Os09g0296800) | 3E-117 |
| PpLhcb9.1 | *Contig7689* | 23:811951-813334 | *C. reinhardtii* | light harvesting complex II protein precursor (Lhcb3) | 2E-54 |
| PpLhcb9.2 | *Contig11747* | 252:201420-203157 | *C. reinhardtii* | light harvesting complex II protein precursor (Lhcb3) | 2E-57 |
| **Added after genome analysis** | | |  |  |  |
| PpLhca5 |  | 284:21545-24365 | *Lycopersicon esculentum* | clone 134347F | 2E-76 |
