# Supplementary figures and images for: In Silico and Biochemical Analysis of Physcomitrella patens Photosynthetic Antenna: Identification of Subunits which Evolved upon Land Adaptation
Source: PLoS One. 2008 Apr 30;3(4):e2033. doi: 10.1371/journal.pone.0002033 (PMC2323573; doi:10.1371/journal.pone.0002033)

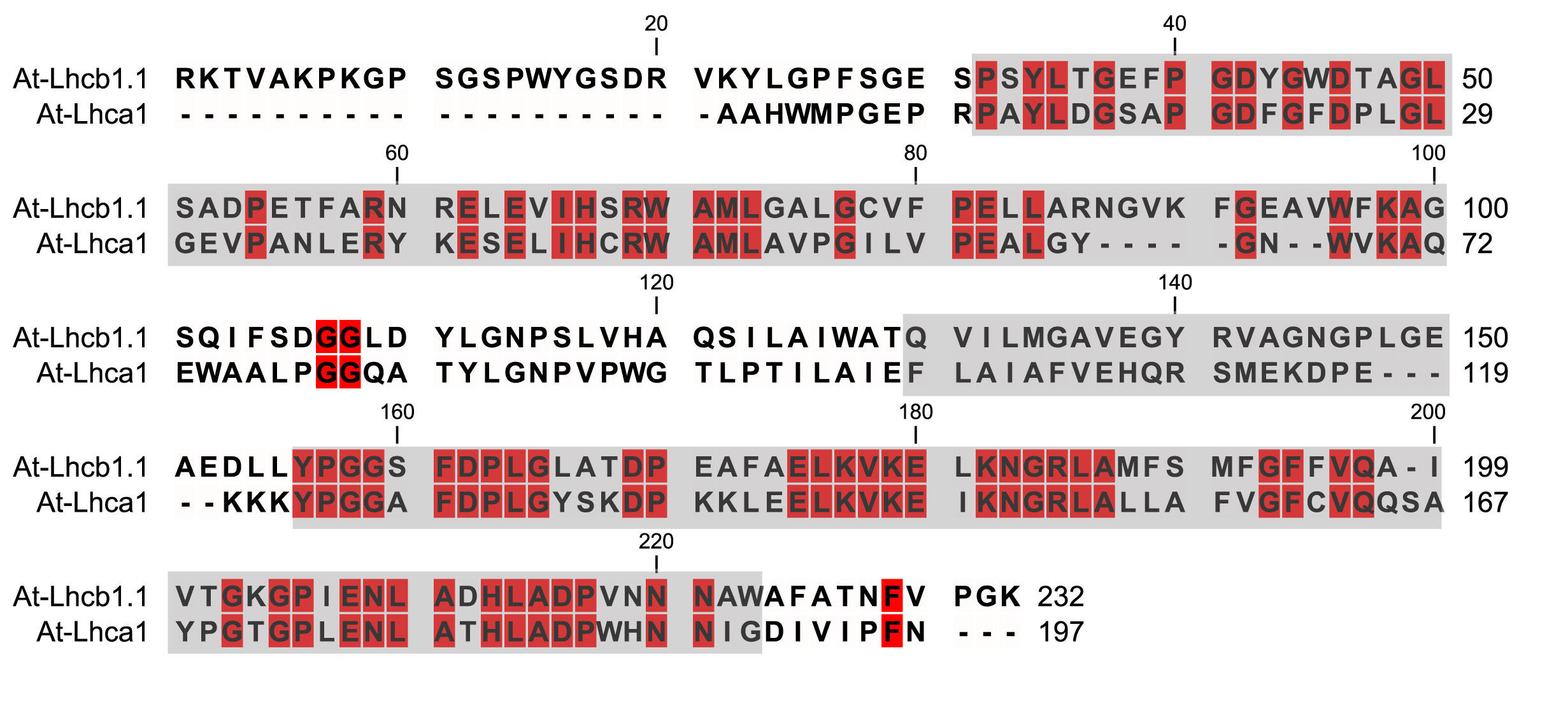

Supplement: Figure S2 — Example of sequence alignments used for generating phylogenetic trees. Only two genes (Lhcb1.1 and Lhca1 from A. thaliana) are shown for clarity. Only the most conserved regions which are indicated in gray were considered for the analysis, as in (Dunford et al., 1999). (8.21 MB TIF) [file pone.0002033.s002.tif]

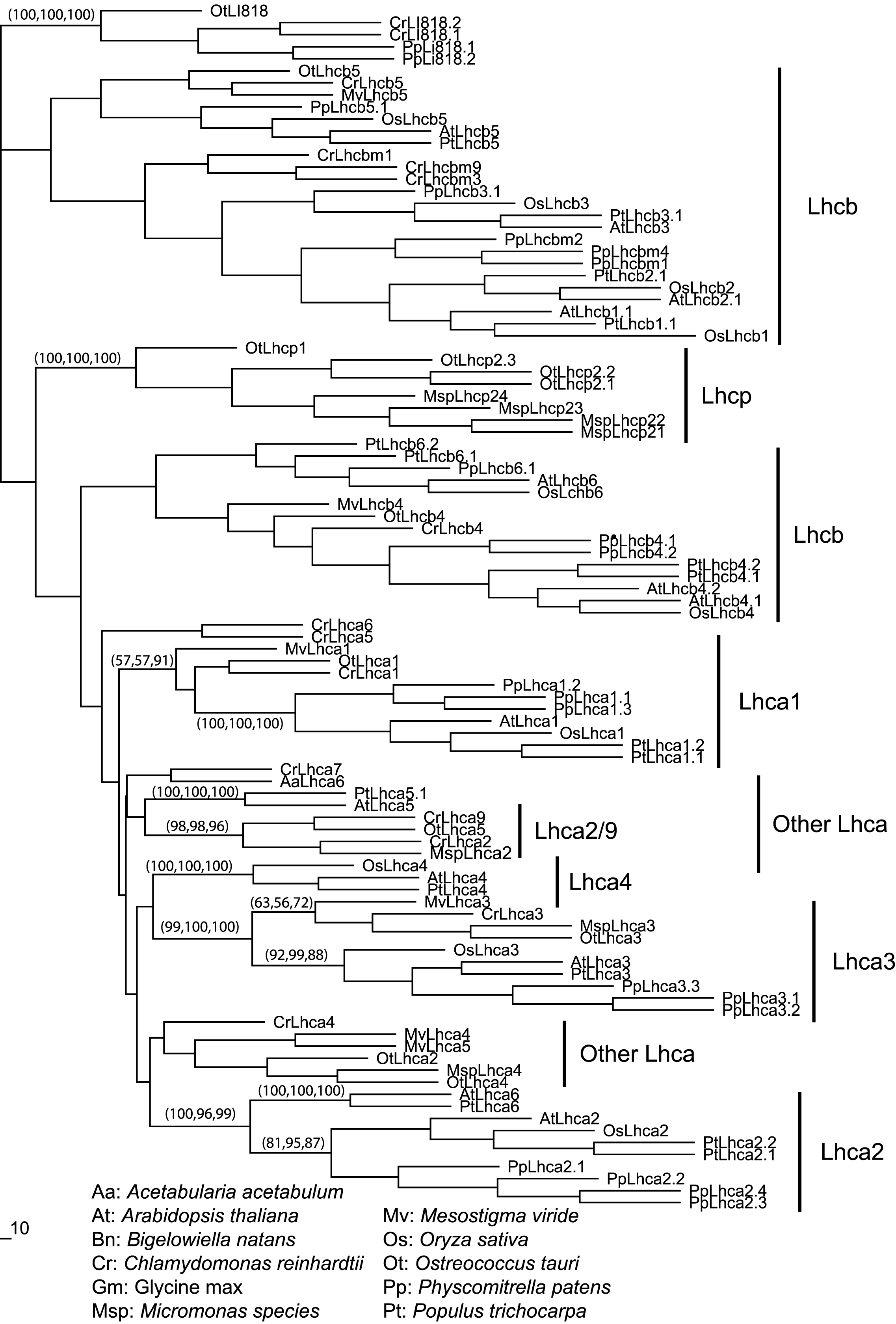

Supplement: Figure S4 — Phylogenetic tree of Lhca antenna proteins. A Phylogenetic tree was generated as described for figure 1. In this case, the number of PSI antenna sequences was increased. A total of 100 sequences were analyzed. Four Li818 sequences from P. patens (Pp), C. reinhardtii (Cr) and O. tauri (Ot) where also included as an external out-group. The tree shown was built using a maximum likelihood approach, but bootstrap values were obtained from maximum likehood, NJD and maximum parsimony approaches, respectively. For clarity, these values are not shown when consistency was poor or when node bootstrap values were not sufficiently significant to discriminate isoforms. (7.64 MB TIF) [file pone.0002033.s004.tif]

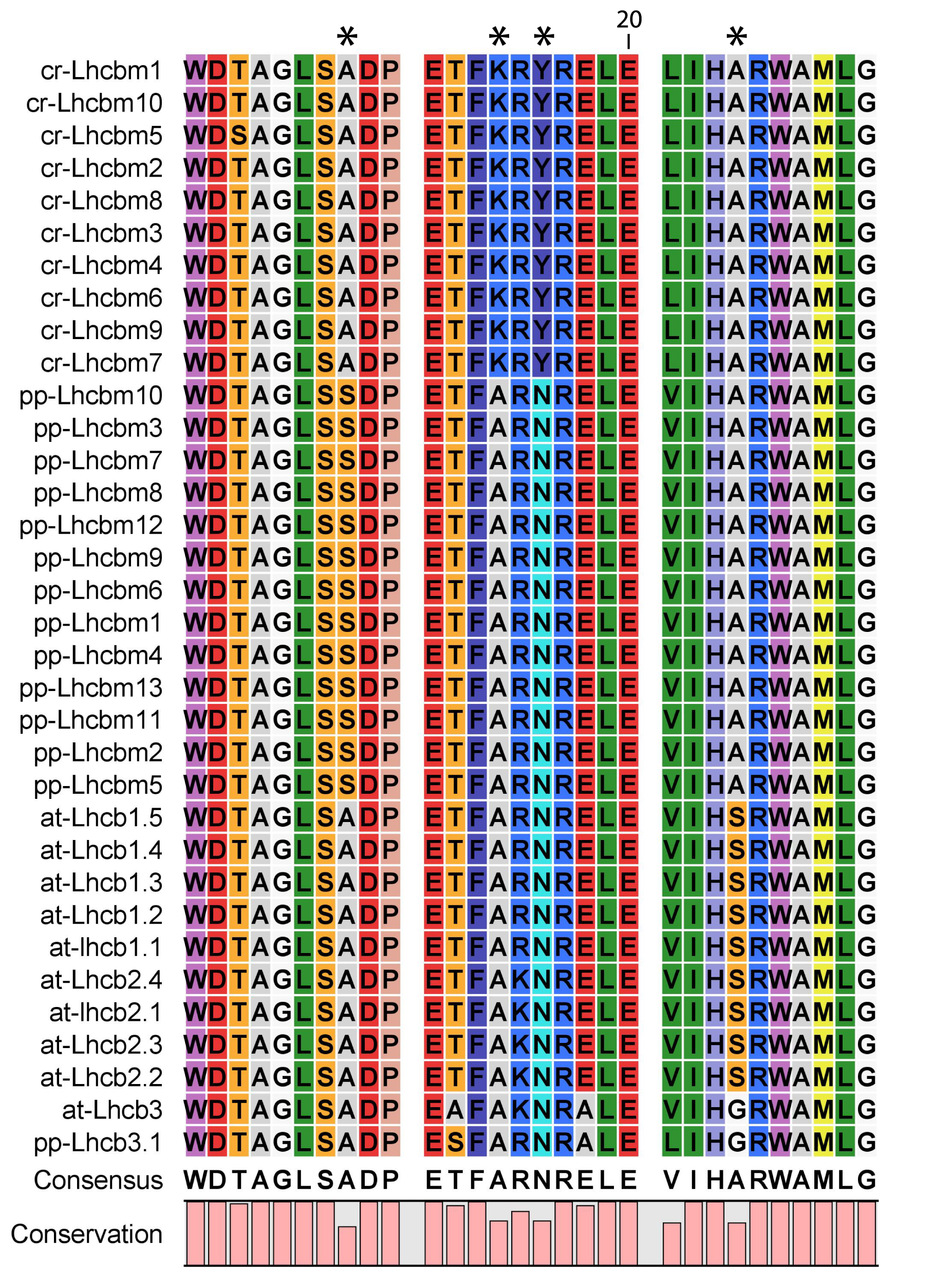

Supplement: Figure S6 — Alignment of PSII major antenna complex polypeptides from C. reinhardtii, P. patens and A. thaliana. The region corresponding to the first transmembrane helix (also called Helix B) is shown. Residues showing species dependent differences are indicated with an asterisk (*). (13.94 MB TIF) [file pone.0002033.s006.tif]
