# Supplementary material for: In Silico and Biochemical Analysis of Physcomitrella patens Photosynthetic Antenna: Identification of Subunits which Evolved upon Land Adaptation
Source: PLoS One. 2008 Apr 30;3(4):e2033. doi: 10.1371/journal.pone.0002033 (PMC2323573; doi:10.1371/journal.pone.0002033)
Supplement: Figure S3 — List of all polypeptide sequences included in the analysis. (0.11 MB DOC) [file pone.0002033.s003.doc]

**Additional Figure S2. List of all polypeptide sequences employed for the analysis.**
