# Supplementary material for: In Silico and Biochemical Analysis of Physcomitrella patens Photosynthetic Antenna: Identification of Subunits which Evolved upon Land Adaptation
Source: PLoS One. 2008 Apr 30;3(4):e2033. doi: 10.1371/journal.pone.0002033 (PMC2323573; doi:10.1371/journal.pone.0002033)
Supplement: Figure S5 — P. patens Lhc-like polypeptides identified in Physcobase. When clear homology was seen the name was derived from the A. thaliana ortholog. In the case of Li818, names were derived from Chlamydomonas nomenclature, since no homologs were found in seed plants. Contig number and the best hit with the tblastn search at NCBI (http://www.ncbi.nlm.nih.gov/BLAST/) are also reported. In a few cases the contig sequence was missing in the database and it was reconstructed by analysis of individual EST clones. Several Lil1 and one Lil2 isoform were not found in the EST database and were retrieved after genome analysis. (0.05 MB DOC) [file pone.0002033.s005.doc]

| **Name** | **Contig** | **Scaffold** | **Organism** | **Annotation** | **E value** |
| --- | --- | --- | --- | --- | --- |
| PpPsbS | *Contig6593* | 241:416472-417974 | *O. sativa* | cDNA clone:OSIGCPI103D12 | 4E-63 |
| PpLi818.1 | *Contig7101* | 213:431042-432258 | *C. reinhardtii* | mRNA for LI818r-3 protein | 5E-70 |
| PpLi818.2 | *Contig1671* | 99:702438-703630 | *C. reinhardtii* | mRNA for LI818r-3 protein | 2E-74 |
| PpLil1.1 (ELIP.1) | *Contig14942* | 78:564138-565229 | *Tortula ruralis* | early light-inducible protein ELIPB | 8E-65 |
| PpLil1.2 (ELIP.2) | *Contig133* | 77:853726-855486 | *Helianthus annuus* | mRNA for homologous early light induced protein | 9E-11 |
| PpLil1.3 (ELIP.3) |  | 255:556066-556917 | *Tortula ruralis* | early light-inducible protein ELIPB | 7E-60 |
| PpLil2.1 (HLIP.1) | *Contig7015* | 33:712243-713768 | *O. sativa* | Os05g0292800 | 9E-14 |
| PpLil3.1 | *Contig11093* | 79:609279-616602 | *A. thaliana* | Lil3.1 (AT4G17600) | 8E-56 |
| PpLil3.2 |  | 336:71921-73368 | *Lycopersicon esculentum* | clone 133525F | 2E-51 |
| PpLil3.3 |  | 21:1047600-1049559 | *A. thaliana* | Lil3.1 (AT4G17600) | 1E-49 |
| PpLil4 (SEP1) | *Contig7368* | 257:580070-581694 | *Solanum lycopersicum* | cDNA, clone: FC19DG10, HTC in fruit | 3E-07 |
| PpLil5 (SEP2) | *Contig7808* | 54:1853915-1855228 | *O. sativa* | Os04g0639200 | 2E-11 |
| PpLil6 |  | 22:2292185-2293914 | O. sativa | OSIGCSN049C08 | 1E-18 |
| PpLil7.1 | *Contig11590* | 31:1544935-1547416 | *Acetabularia acetabulum* | chloroplast photosystem II 22 kDa PsbS protein precursor | 0.008 |
| PpLil7.2 | *Contig8165* | 42:1953897-1956473 | *Acetabularia acetabulum* | chloroplast photosystem II 22 kDa PsbS protein precursor | 0.006 |
| **Added after genome analysis** | | |  |  |  |
| PpLil1.4 (ELIP.4) |  | 140:1-58962 | *Tortula ruralis* | early light-inducible protein ELIPB | 1E-70 |
| PpLil1.5 (ELIP.5) |  | 15:2610038-2610805 | *Tortula ruralis* | early light-inducible protein ELIPB | 4E-70 |
| PpLil1.6 (ELIP.6) |  | 172:741511-742515 | *Tortula ruralis* | early light-inducible protein ELIPB | 2E-82 |
| PpLil1.7 (ELIP.7) |  | 25:2286452-2287295 | *Tortula ruralis* | early light-inducible protein ELIPB | 4E-60 |
| PpLil1.8 (ELIP.8) |  | 239:207874-208926 | *Tortula ruralis* | early light-inducible protein ELIPB | 1E-71 |
| PpLil1.9 (ELIP.9) |  | 196:505412-506415 | *Tortula ruralis* | early light-inducible protein ELIPA | 3E-99 |
| PpLil2.2 (HLIP.2) |  | 12:1090536-1092091 | *O. sativa* | Os05g0292800 | 5E-22 |
